# The Role of Cancer Stem Cell-Derived Exosomes in Cancer Progression

**DOI:** 10.1155/2022/9133658

**Published:** 2022-05-04

**Authors:** Xueting Li, Xinjian Li, Bin Zhang, Baoyu He

**Affiliations:** ^1^Department of Clinical Medicine, Affiliated Hospital of Jining Medical University, Jining Medical University, Jining, Shandong, China; ^2^Department of Nephrology, Affiliated Hospital of Jining Medical University, Jining, Shandong, China; ^3^Department of Laboratory Medicine, Affiliated Hospital of Jining Medical University, Jining Medical University, Jining, Shandong, China

## Abstract

Cancer stem cells (CSCs) represent a small portion of tumor cells with self-renewal ability in tumor tissues and are a key factor in tumor resistance, recurrence, and metastasis. CSCs produce a large number of exosomes through various mechanisms, such as paracrine and autocrine signaling. Studies have shown that CSC-derived exosomes (CSC-Exos) carry a variety of gene mutations and specific epigenetic modifications indicative of unique cell phenotypes and metabolic pathways, enabling exchange of information in the tumor microenvironment (TME) to promote tumor invasion and metastasis. In addition, CSC-Exos carry a variety of metabolites, especially proteins and miRNAs, which can activate signaling pathways to further promote tumor development. CSC-Exos have dual effects on cancer development. Due to advances in liquid biopsy technology for early cancer detection, CSCs-Exos may become an important tool for early cancer diagnosis and therapeutic drug delivery. In this article, we will review how CSC-Exos exert the above effects based on the above two aspects and explore their mechanism of action.

## 1. Introduction

The occurrence of malignant tumors is a multistage and gradual process. Tumor cells gradually undergo malignant transformation through a series of progressive changes. Tumor cells have the characteristics of accelerated growth, enhanced invasiveness and metastasis, and resistance to anticancer drugs [1–3]. Cancer stem cells (CSCs), a subpopulation of cancer cells, have characteristic unlimited proliferation, self-renewal, and multilineage differentiation capabilities. Unlike normal stem cells, CSCs are tumorigenic and have aberrant forms of the normal mechanisms that strictly regulate normal physiology, enabling them to continue to expand and produce abnormally differentiated progeny. Therefore, CSCs promote the progression of multiple malignancies in relation to multiple factors, such as recurrence, metastasis, heterogeneity, multidrug resistance, and radioresistance [4]. CSC-derived exosomes (CSC-Exos) are membranous vesicles secreted by cancer stem cells that carry a variety of biologically active substances, especially proteins and RNAs (microRNAs and lncRNAs), mediating information exchange and material exchange between cells [5]. CSCs-Exos play important roles in the development of cancer due to their biological characteristics; they participate in the occurrence and development of cancer and may represent targets for cancer treatment [6, 7]. Understanding the role of CSC-Exos and their mechanisms can help effectively block related signaling pathways and maximize the benefit of CSC-Exos in cancer treatment.

## 2. The Properties and Biological Functions of CSCs and CSC-Exos in Cancer

The study of CSCs began in 1994, when it was reported that CD34/CD38 cells were human acute myeloid leukemia (AML) stem cells [8]. With the deepening of CSC research in recent years, CSCs have been isolated from almost all solid cancer cell populations. CSCs have characteristic of self-renewal, multidirectional differentiation, and unlimited proliferation capabilities, and they show resistance to chemotherapeutic drugs, strong tumorigenicity, and a strong ability to invade and metastasize [9]. Colombel et al. [10] found that the expression of stem cell-related surface markers such as integrin *α*-2 and integrin *α*-6 was positively correlated with bone metastasis in prostate cancer (PC) patients. CSCs are believed to have a strong invasive ability. Mare et al. [11] found that the ability of breast cancer MCF7 cells pretreated with paclitaxel to form spheroids was enhanced, indicating that paclitaxel enriched breast cancer stem cells, which was consistent with the conclusion that CSCs had chemotherapeutic drug resistance. Although CSCs make up a small portion of cancer cells in cancer tissue, their cancer-forming ability is very strong. This has been repeatedly verified in *in vitro* serum-free spheroid culture experiments and the *in vivo* cancer inoculation experiments in nude mice. Studies have pointed out that 500 to 1000 CSCs are required to form tumors [12]. The above studies provide a more reasonable explanation for the occurrence, invasion, and drug resistance of CSCs in cancers and provide new ideas for cancer therapy.

CSCs-Exos are membranous vesicular bodies secreted by cancer cells. Similar to other exosomes, CSCs-Exos are nanosized vesicles that enable communication between cancer cells and the TME. The formation of CSC-Exos involves four processes: budding, invagination, multivesicular body formation, and secretion [13]. The molecular cargo carried by CSC-Exos is partly derived from the surface of the parent tumor cell. Tumor blasts release millions of exosomes, and CSC-Exos carry oncogenes between cancer cells and normal cells. CSC-Exos also transfer proteins, lipids, and nucleic acids in their functionally active forms. After reaching the recipient cell, CSC-Exos release their contents into specific cells by ligand binding, phagocytosis, and fusion with the plasma membrane and regulate gene expression in recipient cells, thereby determining their behavior [14].

## 3. Cancer-Promoting Effects of CSC-Exos

### 3.1. CSCs-Exos Regulate Cancer Cell Proliferation

**Figure 1:**
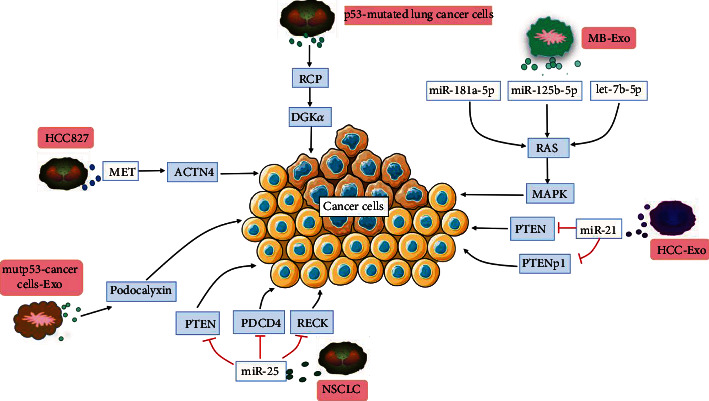
CSC-Exos are involved in cancer cell proliferation. Exosomes secreted from p53-mutated lung cancer cells promote cancer cells proliferation by mediating the RCP/DGK*α* receptor cycling pathway. The production of cancer stem cell-derived exosomes (CSC-Exos) induced by mutated TP53, and these CSC-Exos can regulate the expression levels of podocalyxin and promote cancer cell growth. Hepatocellular carcinoma (HCC) cell-derived exosomes carrying miR-21 promote the proliferation of HCC cells by inhibiting the expression of PTENp1 and PTEN. Exosomes derived from non-small-cell lung cancer (NSCLC) cells carry miR-25 to reduce the expression of the PTEN, PDCD4, and RECK in NSCLC cells, leading to the growth of cancer cells. Medulloblastoma-derived exosomal miRNAs such as miR-181a-5p, miR-125b-5p, and let-7b-5p promote the proliferation of cancer cells via the Ras/MAPK pathway. Medulloblastoma (MB) cell-derived exosomal miRNAs such as miR-181a-5p, miR-125b-5p, and let-7b-5p promote the proliferation and invasion of cancer cells via the Ras/MAPK pathway. Icotinib-resistant human NSCLC (HCC827) cells produce exosomes carrying oncogenic MET mRNAs that mediate NSCLC progression by upregulating alpha-actinin 4 (ACTN4).

The proliferation and apoptosis of cells are controlled by sophisticated genetically programmed regulatory pathways [15]. However, the growth regulation mechanism of cancer cells has been disrupted, and the mutation of tumor regulators (including proto-oncogenes and tumor suppressor genes) is one of the main causes of malignant proliferation of cancer cells [16]. David et al. [17] showed that exosomes secreted from p53-mutated lung cancer cells can promote cancer cell proliferation by affecting the RCP/DGK*α* receptor cycling pathway in vitro. TP53 can be mutated by human papillomavirus (HPV) infection or exposure to carcinogens. Azulay et al. [18] showed that exosomes derived from cancer cells can be induced by mutated TP53 to regulate the expression levels of podocalyxin (PODXL) and promote cancer cell growth in vitro. PTEN is a tumor suppressor gene with dual-specificity phosphatase activity, and its expression is generally reduced in liver cancer. Hepatocellular carcinoma (HCC) cell-derived exosomes (HCC-Exos) carrying the miR-21 molecule promote the proliferation of HCC cells by inhibiting the expression of PTENp1 and PTEN [19]. In addition, Ren et al. [20] have demonstrated that hypoxia-prechallenged exosomes derived from non-small-cell lung cancer (NSCLC) cells carry miR-25; this cargo communicates information with the tumor cell microenvironment, reducing the expression of the PTEN, PDCD4, and RECK genes in NSCLC cells, which leads to the growth of cancer cells. Zhu et al. [21] found that aggressive medulloblastoma cell-derived exosome (MB-Exo) miRNAs such as miR-181a-5p, miR-125b-5p, and let-7b-5p promoted the proliferation and invasion of cancer cells via the Ras/MAPK pathway. Yu et al. [22] showed that icotinib-resistant human NSCLC (HCC827) cells produced exosomes with mRNA encoding MET oncogenes that mediate the progression of NSCLC by upregulating alpha-actinin 4 (ACTN4). CSC-Exos are involved in cancer cell proliferation, as shown in Figure 1.

### 3.2. CSCs-Exos Regulate Angiogenesis in Cancer

**Figure 2:**
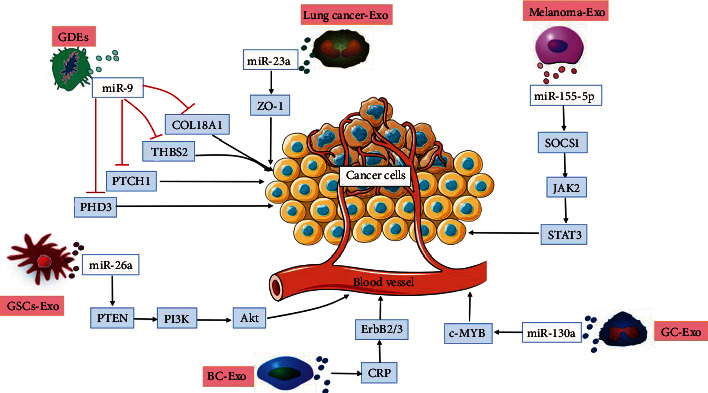
CSC-Exos regulate angiogenesis in cancer. Exosomes derived from lung cancer cells carry miR-23a, which target ZO-1 and promote angiogenesis in cancer. Melanoma cell-derived exosomal miR-155-5p induces a proangiogenic switch of cancer-associated fibroblasts via the SOCS1/JAK2/STAT3 signaling pathway. Exosomes derived from bladder cancer (BC) cells carry CRK, which promotes the expression of ErbB2/3 in BC cells and induces vascular growth in BC. Exosomes derived from gastric cancer (GC) cells contain miR-130a, which induces activation of c-MYB-related angiogenic factors and thereby promotes vascular growth. Glioma cell-derived exosomes (GDEs) carry miR-9, which targets and inhibits COL18A1, THBS2, PTCH1, and PHD3 to promote angiogenesis. miR-26a carried by glioma stem cell-derived exosomes (GSCs-Exo) activates the PI3K/Akt pathway by targeting PTEN to promote angiogenesis in human brain microvascular endothelial cells (HBMECs).

In the process of cancer occurrence and development, tumor cells must activate endothelial cells to promote angiogenesis and provide the necessary substances for their own cell growth [23]. CSC-Exos are involved in angiogenesis in cancer, as shown in Figure 2. The tumor vascular microenvironment greatly promotes the metabolism of tumor cells by promoting extracellular microangiogenesis [24]. Oxygen deficiency is one of the main factors that causes tumor angiogenesis and can promote the expression of exosomal miRNAs [25]. Under hypoxic conditions, exosomes derived from lung cancer cells carry miR-23a, which can target prolyl hydroxylases and the tight junction protein ZO-1, promoting angiogenesis and contributing to cancer progression [26]. Zhou et al. [27] found that melanoma cell-secreted exosomal miR-155-5p induced a proangiogenic switch in cancer-associated fibroblasts via the SOCS1/JAK2/STAT3 signaling pathway. A study [28] demonstrated that exosomes derived from bladder cancer (BC) cells contain CRK, promoting the expression of ErbB2/3 in BC cells and inducing vascular growth in BC. Moreover, studies [29] have shown that exosomes derived from gastric cancer (GC) cells contain miR-130a, and these exosomes carry tumor-derived stimulating factors to induce c-MYB-related angiogenesis, thereby promoting vascular growth. Studies [30] have shown that glioma cell-derived exosomes (GDEs) can transport miR-9, which targets and inhibits COL18A1, THBS2, PTCH1, and PHD3 to promote angiogenesis. Moreover, overexpression of miR-26a in glioma stem cell-derived exosomes (GSC-Exos) activates the phosphatidylinositol 3-kinase (PI3K)/Akt pathway by targeting PTEN in vitro, thereby promoting the proliferation and angiogenesis of human brain microvascular endothelial cells (HBMECs) [31].

### 3.3. CSCs-Exos Promote Cancer Metastasis and Infiltration

**Figure 3:**
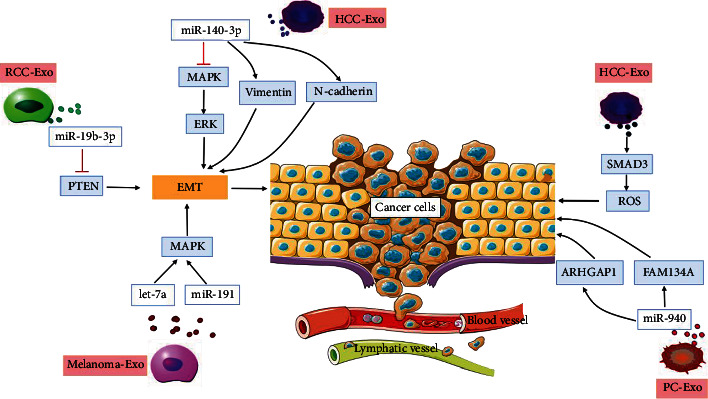
CSC-Exos are involved in cancer metastasis and infiltration. Exosomes derived from renal clear carcinoma (RCC) stem cells carry miR-19b-3p, which inhibits the expression of PTEN in the cell and mediates epithelial-mesenchymal transition (EMT) to promote cancer metastasis. Melanoma cell-derived exosomes carry let-7a and miR-191, which activate MAPK signaling to mediate EMT to promote metastasis. miR-140-3p in exosomes secreted by hepatocellular carcinoma (HCC) cells can inhibit MAPK/ERK pathway activity and increase the expression of vimentin and N-cadherin to induce EMT and tumor metastasis. Primary hepatocellular carcinoma (HCC) cell-derived exosomes facilitate metastasis by regulating adhesion of circulating tumor cells and inducing reactive oxygen species (ROS) production via SMAD3 signaling in liver cancer. Exosomes secreted by prostate cancer (PC) cells contain miR-940, which acts on ARHGAP1 and FAM134A in osteoblasts to promote the formation of a bone metastatic microenvironment.

Exosomes can directly promote cancer cell metastasis and infiltration because proteins, lipids, and RNA from exosomes act on recipient cells, weakening the adhesion between cells [32]. Tumor cell migration is fundamental to the metastasis and infiltration of surrounding normal tissue by cancer cells. Epithelial-mesenchymal transition (EMT) is a key step in cancer metastasis and infiltration. CSC-Exos act as transporters of EMT initiation signals and transfer such signals to tumor cells, causing cancer metastasis and infiltration [33]. For example, exosomes derived from renal clear carcinoma (RCC) stem cells carried miR-19b-3p, which inhibited the expression of PTEN in the cell, thereby inducing EMT [34]. Melanoma cell-derived exosomes promoted a phenotypic switch of primary melanocytes via autocrine signaling. We found that molecules carried in exosomes (let-7a and miR-191) activate MAPK signaling to mediate the process of EMT and promote metastasis [35]. miR-140-3p in HCC-Exos can inhibit MAPK/ERK pathway activity and increase muscle activity, increasing the expression of vimentin and N-cadherin and ultimately inducing the occurrence of EMT and tumor metastasis [36]. Therefore, miRNAs contained in exosomes participate in EMT regulation and can enable malignant tumor cells derived from epithelial cells to acquire stronger invasion and migration capabilities. Fu et al. clarified that primary HCC-Exos facilitated metastasis by regulating the adhesion of circulating tumor cells and inducing reactive oxygen species (ROS) production via SMAD3 signaling in a paracrine and autocrine manner [37]. Hashimoto et al. [38] found that exosomes secreted by PC cells contain miR-940, which could act on ARHGAP1 and FAM134A in osteoblasts to promote the formation of the bone metastatic microenvironment, which was conducive to the distant metastasis of PC. CSC-Exos are involved in cancer metastasis and infiltration, as shown in Figure 3.

### 3.4. CSCs-Exos Regulate Cancer Cell Evasion of Immune Surveillance

**Figure 4:**
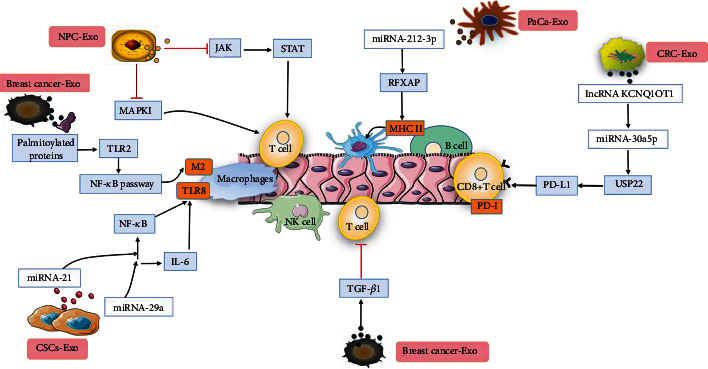
CSC-Exos regulate cancer cells to evade immune surveillance. MiRNAs contained in nasopharyngeal carcinoma (NPC) cell-derived exosomes downregulate the MAPKI and JAK/STAT pathways to impair T-cell proliferation, differentiation, and cytokine secretion. miRNA-21 and miRNA-29a can bind to Toll-like receptor 8 (TLR8) on the surface of tumor-associated macrophages through the action of CSC-Exos, triggering the NF-*κ*B pathway and the secretion of interleukin-6 (IL-6) to induce evasion of immune surveillance. Exosomes derived from pancreatic cancer (PaCa) cells contain miR-212-3p, which inhibits the expression of regulatory factor X-associated protein (RFXAP), resulting in a decrease in the expression of MHC II molecules (molecules on the surface of B cells) and induction of immune tolerance. Breast cancer-derived exosomes inhibit the proliferation of T cells through TGF-*β*1, interfering with normal immune system function. The lncRNA KCNQ1OT1 loaded in colorectal cancer cell-derived exosomes (CRC-Exos) mediates the miR-30a-5p/USP22 pathway to regulate the ubiquitination of PD-L1 and inhibits the CD8+ T-cell response, thereby promoting colorectal cancer cell evasion of immune surveillance. Macrophage immunomodulation by breast cancer cell-derived exosomes requires Toll-like receptor 2-mediated activation of the NF-*κ*B pathway, which induces pro-inflammatory activity of distant macrophages in cancer progression.

The immune system can resist attacks from external invaders such as bacteria and viruses. Upon recognition of invaders, the immune system will activate various chemical and physiological processes to form an immune response. However, many cancer cells have multiple immune escape mechanisms, such as avoiding cytotoxic cell recognition by directly damaging the function of antigen-presenting cells or cytotoxic cells and activating immunosuppressive cells [39]. Moreover, studies [40] have shown that CSCs release exosomes containing RNAs (microRNAs and lncRNAs) and proteins to participate in evasion of immune surveillance. CSC-Exos are involved in regulating cancer cell evasion of immune surveillance, as shown in Figure 4. Research has shown that exosomes can participate in the evasion of immune surveillance through T cells [41]. Yin et al. [42] found that CSC-Exos were rich in immunosuppressive proteins, such as programmed death-ligand 1 (PD-L1). PDL-1 is highly expressed on the surface of tumor cells and binds to its receptor on the surface to inhibit the activation of T cells, causing cancer cells to evade antitumor immunity. Ye et al. [43] found that miRNAs contained in nasopharyngeal carcinoma cell-derived exosomes downregulated the MAPKI and JAK/STAT pathways to impair T-cell proliferation, differentiation, and cytokine secretion. Cancer-associated fibroblasts (CAFs) are a stromal cell population with various cells of origin and phenotype and functional heterogeneity [44]. They play an important role in the development of cancer. The tumor immune microenvironment (TIME) in tumor pancreatic islets is mainly composed of different immune cell groups, which are highly correlated with the antitumor immune status in the TME [45]. Mao et al. [46] found that CAF exosomes carried various cytokines, growth factors, chemokines, and other effector molecules and interacted with tumor-infiltrating immune cells and other immune components in the TIME to form an immunosuppressive TME, which enables cancer cells to escape the immune system. TGF-*β* is one of the major immunosuppressive cytokines, and natural killer (NK) cells are inhibited by TGF-*β*1 loaded in blast-derived exosomes [47]. Moreover, breast cancer cell-derived exosomes also inhibited the proliferation of T cells through TGF-*β*1, interfering with normal immune system function and thereby promoting tumor development [48]. Fabbri et al. [49] found that miRNA-21 and miRNA-29a carried in CSC-Exos can bind to Toll-like receptor 8 (TLR8) on the surface of tumor-associated macrophages (TAMs), triggering the NF-*κ*B pathway and the secretion of interleukin-6 (IL-6). CSC-Exos can also interfere with the immune system in multiple ways and drive cancer cells to evade immune surveillance. Exosomes derived from pancreatic cancer (PaCa) cells had high levels of miR-212-3p, which inhibited the expression of regulatory factor X-associated protein (RFXAP), resulting in a decrease in the expression of MHC II molecules and inducing immune tolerance [50]. Xian et al. found that the tumor-promoting effect of the lncRNA KCNQ1OT1 occurred through autocrine effects of colorectal cancer cell-derived exosomes (CRC-Exos), which mediated the miR-30a-5p/USP22 pathway to regulate the ubiquitination of PD-L1 and inhibit the CD8+ T-cell response, thereby promoting colorectal cancer development [51]. Growing evidence links tumor progression with the activity of various immune cells, such as macrophages. Chow et al. [52] found that palmitoylated proteins present on the surface of breast cancer cell-derived exosomes contributed to Toll-like receptor 2-mediated activation of the NF-*κ*B pathway to induce the pro-inflammatory activity of distant macrophages in cancer progression.

### 3.5. CSC-Exos Play a Role in Regulating the TME

**Figure 5:**
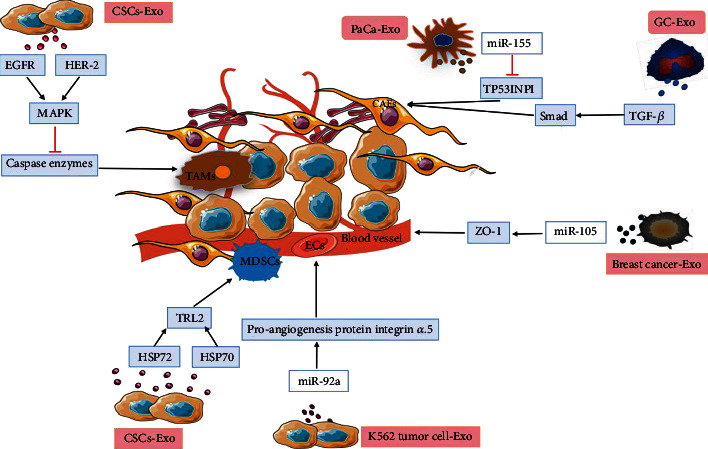
CSC-Exos play a role in the regulation of the tumor microenvironment (TME). Exosomes derived from pancreatic cancer (PaCa) cells are rich of miR-155 and promote the differentiation of fibroblasts into cancer-associated fibroblasts (CAFs) by downregulating the level of TP53INP1 protein in fibroblasts. Proteins carried by CSC-Exos, such as HSP72 and HSP70, target downstream Toll-like receptor 2 (TLR2) to activate myeloid-derived suppressor cells (MDSCs) to promote the formation of the TME. miR-92a loaded in exosomes derived from K562 tumor cells interacts with the proangiogenic protein integrin *α*5, causing endothelial cell (EC) migration and primitive vascular lumen formation. Breast cancer-derived exosomes contain miR-105, which downregulates the expression of ZO-1 to increase the permeability of tumor blood vessels in the TME. Gastric cancer cell-derived exosomes (GC-Exos) carry TGF-*β*, which induces human umbilical cord mesenchymal stem cells (hucMSCs) to differentiate into CAFs through the TGF-*β*/Smad pathway. Epidermal growth factor receptor (EGFR) and human epidermal growth factor receptor (HER-2) riched in CSC-Exos activate the MAPK signaling pathway of monocytes and inhibit the cleavage of caspases, which contributes to the formation of tumor-associated macrophages (TAMs).

The cause of death in patients with cancer is often systemic multiple organ failure caused by widespread metastasis. CSC-Exos regulate the formation of the microenvironment before the arrival of cancer cells, helping cancer cells metastasize and infiltrate the surrounding tissues [53]. The TME is a steady-state environment composed of tumor cells, TAMs, CAFs, myeloid-derived suppressor cells (MDSCs), vascular endothelial cells, and extracellular matrix (ECM) [54] and fosters the occurrence and development of tumors. The components in the TME can directly secrete metabolites (such as IL-6, FGF-2, PDGF, MMPs, CXC12, VEGF, FGF, IL8/CXCL8, and PDGF-C) that induce tumor metastasis and tumor cell proliferation. In response, tumor cells interact with the TME by secreting growth factors (such as FGF-2 and PDGF) and chemokines (such as CXCL12) and induce mechanical stress that ultimately leads to cancer progression [55]. CAFs are the main cellular components of the TME. They can secrete a large number of cytokines and chemokines to participate in tumor growth and metastasis. Studies have shown that CSC-Exos can induce the generation of CAFs, which may be related to TGF-*β*. A study [56] found that GC-Exos carry TGF-*β*, which can induce human umbilical cord mesenchymal stem cells (hucMSCs) to differentiate into CAFs through the TGF-*β*/Smad pathway. Pang et al. [57] found that exosomes derived from PaCa cells were rich in miR-155 and promoted the differentiation of fibroblasts into CAFs by downregulating the level of TP53INP1 protein in fibroblasts. Immunosuppression is one of the main features of the TME. TAMs and MDSCs play an important role in the TME. CSC-Exos usually carry epidermal growth factor receptor (EGFR) and human epidermal growth factor receptor (HER-2). These receptors can activate the MAPK signaling pathway of monocytes and inhibit the cleavage of caspase enzymes, which is conducive to the formation of TAMs, and proteins carried by CSC-Exos, such as HSP72 and HSP70, can target downstream Toll-like receptor 2 (TLR2) to activate myeloid-derived suppressor cells (MDSCs) and promote the formation of the TME [58]. CSC-Exos contain a large number of miRNAs related to angiogenesis. miR-92a contained in exosomes derived from K562 tumor cells interacts with the proangiogenic protein integrin *α*5, causing endothelial cell migration and primitive vascular lumen formation [59]. Breast tumor cell-derived exosomes contained miR-105, which downregulated the expression of the endothelial tight junction protein ZO-1, directly affecting endothelial tight junctions and increasing the permeability of tumor blood vessels [60]. CSC-Exos are involved in the regulation of the TME, as shown in Figure 5.

### 3.6. The Role of CSCs-Exos in Cancer Chemoresistance

Chemoresistance has become the largest obstacle in cancer treatment. CSC-Exos participate in the development of chemoresistance through multiple mechanisms. The specific manifestations are as follows: (1) By acting on recipient cells, CSC-Exos can induce the formation of premetastatic niches and reprogram the cell cycle and apoptosis genes of recipient cells [61]. Exosomes derived from HER2+ breast cancer cells carry lncRNA-SNHG14, which can induce apoptosis and trastuzumab resistance by targeting the B-cell lymphoma-2 gene (Bcl-2)/BAX pathway [62]. Fornari F et al. [63] found that miR-221 carried by exosomes derived from HCC cells directly targeted caspase-3, thus promoting cancer cell apoptosis and increasing the resistance of HCC cells to sorafenib. (2) CSC-Exos reduce the effective utilization of drugs by increasing drug efflux, reducing cell lysis, and isolating cytotoxic drugs. ATP-binding cassette transporter (ABC) proteins are ATP-driven pumps responsible for transferring drugs to the outside of the cell; examples include P-glycoprotein (Pgp, encoded by the ABCB1 gene) and MDR-associated protein 1 (MRP1, encoded by the ABCC1 gene), which play major roles in chemoresistance [64]. LV et al. [65] found that chemoresistant breast cancer cells can transmit P-gp to sensitive cells through CSC-Exos, thereby making sensitive cells resistant to chemotherapy. Moreover, studies have found that exosomes derived from PC will transfer docetaxel from the cell through the MDR-1/P-gp pathway, increasing the chemoresistance of cancer cells [66]. (3) CSC-Exos can transfer a chemoresistance phenotype from chemoresistant cells to chemosensitive cells and decrease drug sensitivity in chemosensitive cells. Studies [67] have shown that CSC-Exos have the ability to horizontally transfer drug resistance by transmitting genetic material, which can make sensitive cells resistant. Hepatoblastoma cell-derived exosomes can induce Huh6 cells to overexpress interleukin-34 (IL-34) via Brd4 signaling and induce drug resistance in an autocrine manner [68]. Hu et al. [69] found that exosomes secreted by intestinal tumor cells stabilize *β*-catenin and induced nuclear translocation, activating the Wnt/*β* signaling pathway, which makes colorectal cancer cells resistant to 5-FU and oxaliplatin. Studies [70] have shown that exosomes derived from triple-negative breast cancer cells can induce docetaxel and gemcitabine resistance in nontumorigenic breast cells by upregulating the PI3K/AKT, MAPK, and HIF1A signaling pathways.

### 3.7. The Role of CSC-Exos in Autophagy of Cancer

Autophagy is a method of eliminating damaged and misfolded proteins, protein aggregates, damaged organelles, and intracellular pathogens [71]. However, under stress conditions such as hypoxia, nutrient deprivation, organelle damage, and protein damage, exosome-based autophagy networks crosstalk and contribute to the development of cancer by increasing drug resistance and metastasis [72]. Dutta et al. showed that exosomes from breast cancer cells can induce autophagic flux in mammary epithelial cells *in vitro*, stimulate the production of large amounts of ROS, induce autophagy-related tumor growth-promoting factor secretion from recipient cells, and accelerate cancer progression [73]. In addition, autophagy can exhibit prometastatic properties in the early stages of cancer development, promoting cancer cell survival and migration to secondary tissues. Exosomes derived from breast cancer cells can activate autophagy-related genes, including LC3 and Beclin-1, to promote the proliferation, motility, and invasion of breast cancer cells [74]. Exosomes derived from breast cancer cells carried prolyl carboxypeptidase (PRCP), glucose-regulated protein 78 (GRP78), and lncRNA H19, which mediated selective estrogen receptor modulator (SERM) and resistance to enzyme inhibitors [75], the aforementioned drugs induce autophagy, which is associated with drug resistance. The above research results consistently demonstrate that crosstalk between exosome biogenesis and autophagy pathways orchestrates intratumoral communication.

## 4. Exosomal Contents as Biomarkers in Cancer

Exosomes have many natural advantages; for example, exosomes protect nucleic acid substances and prevent them from being degraded, and the formation of exosomes is closely related to the state of parent cells. Exosome content is more specific than traditional tumor markers [76]. Exosomes are widely present in a variety of body fluid samples, and tumor monitoring based on exosomes can be used to detect changes in molecular markers over time during the development of the disease [77]. Such markers are easier to monitor, and the samples are easier to collect; as such, exosomes can be used for the early diagnosis of clinical tumors. Studies [78] have shown that the composition of miRNAs and proteins secreted by exosomes in the body fluids of patients with liver cancer, lung cancer, PC, BC, and other malignant tumors is quite different from that of normal human fluids; thus, these contents can be used as specific markers for some tumor types. Exosome content is helpful for the diagnosis and prognosis prediction of disease (see Tables 1 and 2 for details).

## 5. Cancer-Inhibiting Effects of Exosomes

### 5.1. Direct Antitumor Effects of CSC-Exos

CSC-Exos may be viewed as a “double-edged sword” and are closely related to cancer [104]. CSCs-Exos play an important role in the occurrence and progression of cancer; however, exosomes can also be used in cancer diagnosis and treatment. Studies [105] have shown that CSC-Exos can have a direct antitumor effect by inhibiting the progression of disease. Zhang et al. [106] found that the level of miR-320a in CAF-derived exosomes of HCC patients was significantly reduced, and *in vivo* experiments further revealed that miR-320a directly interacted with the downstream target protein PBX3, inhibiting the proliferation and migration of HCC cells by inhibiting the MAPK pathway. CSCs-Exos exerted potential antitumor effects by inducing cancer cell apoptosis. For example, exosomes secreted by PaCa cells can increase the expression of Bcl-2-related X protein (Bax) and reduce the expression of Bcl-2, inhibiting the PI3K/Akt signaling pathway to drive tumor cell apoptosis [107]. Exosomes derived from the plasma of BC patients carried the lncRNA PTENP1, which increased cell apoptosis and the invasion and migration ability of BC cells [108]. Xu et al. [109] found that exosomes from gastric CAFs carried miR-139, which inhibited the progression and metastasis of gastric cancer cells by reducing MMP11 in the TME. Exosomal miR-9 from nasopharyngeal carcinoma cells inhibited the formation of endothelial tubes and the migration of endothelial cells by inhibiting the MDK/PDK/AKT signaling pathway [110]. The above research shows that CSC-Exos not only can be used as markers of cancer to facilitate early diagnosis but also have unlimited potential in the treatment of cancer.

### 5.2. CSC-Exos as Drug Carriers

Targeted therapy has become an increasingly widely used therapeutic method for cancer [111]. In recent years, a variety of synthetic targeted drug delivery systems have been developed and introduced into the market. However, due to inefficiency, cytotoxicity, and/or immunogenicity, the application of such systems is limited [112]. Meanwhile, due to their unique composition, CSC-Exos have become new carriers for the therapeutic delivery of drugs [113]. The lipid bilayer maintains the integrity of exosomes and stabilizes their biological activity, which makes it easier for them to pass through biological barriers in the human body [114]. The proteins on the surface of exosomes enhance their recognition and targeting capabilities, and the abundant RNA species promote their regulation of receptor cell transcription and translation [115]. The small size, low immunogenicity, long half-life, good permeability, and good biocompatibility of CSC-Exos make them one of the best choices for targeted cancer treatment via drug delivery vehicle [116]. To date, drugs have been transported in a variety of ways, such as exosomal incubation, electroporation, ultrasonic treatment, extrusion, freeze–thaw cycle-based administration, and saponin-based administration [117]. Pan et al. [118] embedded a nanoparticle called PMA/Fe-HSA@DOX into the urine exosomes of PC patients to create a bionic Exo-PMA/Fe-HSA@DOX Trojan nanocarrier. High expression of the membrane protein antigen CD47 on exosomes can reduce the clearance rate of new psychoactive substances in circulating microparticles, making it easier for nanocarriers to enter the cell, and catalyze endogenous H2O2 in cells to produce toxic ·OH. Toxic ·OH and low-dose doxycycline (DOX) are effective in synergistically inducing apoptosis of cancer cells and inhibiting EGFR and its downstream AKT/NF-*κ*B/I*κ*B signaling pathway, enhancing the effect of cancer treatment. CSC-Exos can also be combined with superparamagnetic nanoparticles to form exosome-superparamagnetic nanoparticle (SMNC-EXO) complexes. Accumulation of the hydrophobic drug doxorubicin in the tumor site can help to kill cancer cells [119]. Studies [120] have shown that exosomes purified from MCF-7 human cancer breast cells can be used with ultrasonic electroporation technology to induce anticancer effects by silencing lncRNAs and miRNAs related to the HER2 gene in recipient cells. Yong et al. [121] found that biocompatible biomimetic porous silicon nanoparticles (PSiNPs) of CSC-Exos could be developed as drug carriers for targeted cancer chemotherapy. Exosome-encapsulated doxorubicin PSiNPs (DOX@E-PSiNPs) can be endocytosed by CSC-Exos and targeted for export to dense tumor cells to kill cancer cells. Doxorubicin can be housed inside exosomes derived from certain fibrosarcoma cells (HT1080) by membrane extrusion to form a complex called D-exos. After 12 hours, doxorubicin is removed from D-exos in HT1080 cells, isolated from the exosomes, and distributed in the place where cancer cells gather [122].

## 6. Conclusion

CSC-Exos represent novel tools for intercellular information exchanges and sources of noninvasive tumor markers and participate in the occurrence and development of a variety of cancers, indicating their substantial application value in cancer. To date, the study of CSC-Exos has led to new avenues for the study of mechanisms such as the immune response, immune escape, immune tolerance, tumor invasion, and metastasis and has provided new ideas for targeted tumor therapy. Although reports and studies of CSC-Exos have emerged widely in recent years, there is much that remains unknown about exosomes, and their specific mechanism of action in the TME has not been clarified and needs further research. However, it is believed that an increased understanding of the various mechanisms of exosomes will reveal that exosomes are better options for clinical treatment strategies, and they can be used to develop new methods of tumor treatment and bring benefit to cancer patients.

## Figures and Tables

**Table 1:** CSC-Exo-derived RNAs acting as biomarkers in cancers.

Cancer type	Biomarkers	Level trend	Reference
Hepatic cell cancer	miR-222, miR-221, miR-23, miR-665, miR-224, miR-103, miR-181c, miR-181a, miR-26a	↑	[79–81]
Lung cancer	miR-181b-5p, miR-21-5p, miR-378a, miR-379, miR-139-5p, miR-200b-5p, miR-151a-5p, miR-30a-3p, miR-200b-5p	↑	[82]
Lung cancer	miR-20b, miR-30e-3p	↓	[83]
Pancreatic cancer	miR-1246, miR-4644, miR-3976, miR-4306, miR-21, miR-155, miR-17-5p, miR-196a	↑	[84, 85]
Colorectal cancer	miR-23a, miR-1246, miR-21, miR-6803-5p, miR-139-3p, miR-145-3p	↑	[86]
Prostate cancer	miR-375, miR-141, miR-200b, miR-516a-3p, miR-21, miR-221	↓	[87]
Prostate cancer	miR-let-7a, miR-let-7e, miR-24, miR-26b, miR-30c, miR-145, miR-155	↑	[88]
Breast cancer	miR-373, miR-155, miR-21, miR-1246, miR-106a363, miR-101, miR-327	↑	[89–91]
Ovarian cancer	miR-21, miR-141, miR-203, miR-205, miR-214, miR-92, miR-93	↑	[92, 93]
Breast cancer	miR-187, miR-18a, miR-25, miR-142-3p, miR-140-5p, miR-204, miR-126, miR-182, miR-199a	↑	[94, 95]
Melanoma	miR-101, miR-182, miR-221, miR-222, miR-106-363, miR-106a, miR-92, miR-196, miR-21, miR-156, miR-214, miR-30b, miR-30d, miR-532-5p	↑	[96]
Melanoma	miR-31, miR-125b, miR-148a, miR-211, miR-193b, miR-196a-1, miR-196a-2, miR-203	↓	[96]

**Table 2:** CSC-Exo-derived proteins acting as biomarkers in cancer.

Cancer type	Biomarkers	Level trend	Reference source
Glioblastoma	HSP, NANOGP8, (EGFR), EGFRv11I, IDHl	↑	[97]
Pancreatic cancer	ZIP4, GPC1	↑	[98]
Colorectal cancer	CD147, CPNE3	↑	[99]
Prostate cancer	CD276, HSP72, PSA, PSMA, ITGA3, ITGB1	↑	[100]
Gastric cancer	AHSG, FGA, APOA-I	↑	[101]
Ovarian cancer	TUBB3, EpCAM, CLDN3, PCNA, EGFR, APOE	↑	[102]
Breast cancer	Mucin-1, CEACAM-5, EPS8L2, moesin, K17	↑	[103]

## References

[1] Mullard A. (2020). Addressing cancer's grand challenges. *Nature Reviews. Drug Discovery*.

[2] Wu X., Li J., Gassa A. (2021). Circulating tumor DNA as an emerging liquid biopsy biomarker for early diagnosis and therapeutic monitoring in hepatocellular carcinoma. *International Journal of Biological Sciences*.

[3] Campos-Carrillo A., Weitzel J. N., Sahoo P. (2020). Circulating tumor DNA as an early cancer detection tool. *Pharmacology & Therapeutics*.

[4] Papaccio F., Paino F., Regad T., Papaccio G., Desiderio V., Tirino V. (2017). Concise review: cancer cells, cancer stem cells, and mesenchymal stem cells: influence in cancer development. *Stem Cells Translational Medicine*.

[5] Annett S., Robson T. (2018). Targeting cancer stem cells in the clinic: current status and perspectives. *Pharmacology & Therapeutics*.

[6] Yang L., Shi P., Zhao G. (2020). Targeting cancer stem cell pathways for cancer therapy. *Signal Transduction and Targeted Therapy*.

[7] Whiteside T. L. (2016). Tumor-derived exosomes and their role in cancer progression. *Advances in Clinical Chemistry*.

[8] Lapidot T., Sirard C., Vormoor J. (1994). A cell initiating human acute myeloid leukaemia after transplantation into SCID mice. *Nature*.

[9] Najafi M., Farhood B., Mortezaee K. (2019). Cancer stem cells (CSCs) in cancer progression and therapy. *Journal of Cellular Physiology*.

[10] Colombel M., Eaton C. L., Hamdy F. (2012). Increased expression of putative cancer stem cell markers in primary prostate cancer is associated with progression of bone metastases. *The Prostate*.

[11] de la Mare J.-A., Sterrenberg J. N., Sukhthankar M. G. (2013). Assessment of potential anti-cancer stem cell activity of marine algal compounds using an in vitro mammosphere assay. *Cancer Cell International*.

[12] Weissman I. L. (2005). Normal and neoplastic stem cells. *Novartis Foundation Symposium*.

[13] Zhou Y., Zhang Y., Gong H., Luo S., Cui Y. (2021). The role of exosomes and their applications in cancer. *International Journal of Molecular Sciences*.

[14] Lopatina T., Gai C., Deregibus M. C., Kholia S., Camussi G. (2016). Cross talk between cancer and mesenchymal stem cells through extracellular vesicles carrying nucleic acids. *Frontiers in Oncology*.

[15] Neutzner A., Li S., Xu S., Karbowski M. (2012). The ubiquitin/proteasome system-dependent control of mitochondrial steps in apoptosis. *Seminars in Cell & Developmental Biology*.

[16] Kontomanolis E. N., Koutras A., Syllaios A. (2020). Role of oncogenes and tumor-suppressor genes in carcinogenesis: a review. *Anticancer Research*.

[17] Novo D., Heath N., Mitchell L. (2018). Mutant p53s generate pro-invasive niches by influencing exosome podocalyxin levels. *Nature Communications*.

[18] Azulay E. E., Cooks T., Elkabets M. (2020). Potential oncogenic roles of mutant-p53-derived exosomes in the tumor-host interaction of head and neck cancers. *Cancer Immunology, Immunotherapy*.

[19] Cao L.-Q., Yang X.-w., Chen Y.-b., Zhang D.-w., Jiang X.-F., Xue P. (2020). Correction to: Exosomal miR-21 regulates the TETs/PTENp1/PTEN pathway to promote hepatocellular carcinoma growth. *Molecular Cancer*.

[20] Ren W., Hou J., Yang C. (2019). Extracellular vesicles secreted by hypoxia pre-challenged mesenchymal stem cells promote non-small cell lung cancer cell growth and mobility as well as macrophage M2 polarization via miR-21-5p delivery. *Journal of experimental & clinical cancer research: CR*.

[21] Zhu L.-Y., Wu X.-Y., Liu X.-D. (2020). Aggressive medulloblastoma-derived exosomal miRNAs promote in vitro invasion and migration of tumor cells via Ras/MAPK pathway. *Journal of Neuropathology and Experimental Neurology*.

[22] Yu Y., Abudula M., Li C., Chen Z., Zhang Y., Chen Y. (2019). Icotinib-resistant HCC827 cells produce exosomes with mRNA MET oncogenes and mediate the migration and invasion of NSCLC. *Respiratory Research*.

[23] Demircioglu F., Hodivala-Dilke K. (2016). *α*v*β*3 Integrin and tumour blood vessels -- learning from the past to shape the future. *Current Opinion in Cell Biology*.

[24] Tan H. W., Xu Y. M., Qin S. H., Chen G. F., Lau A. T. Y. (2021). Epigenetic regulation of angiogenesis in lung cancer. *Journal of Cellular Physiology*.

[25] Goradel N. H., Mohammadi N., Haghi-Aminjan H., Farhood B., Negahdari B., Sahebkar A. (2019). Regulation of tumor angiogenesis by microRNAs: state of the art. *Journal of Cellular Physiology*.

[26] Hsu Y. L., Hung J. Y., Chang W. A. (2017). Hypoxic lung cancer-secreted exosomal miR-23a increased angiogenesis and vascular permeability by targeting prolyl hydroxylase and tight junction protein ZO-1. *Oncogene*.

[27] Zhou X., Yan T., Huang C. (2018). Melanoma cell-secreted exosomal miR-155-5p induce proangiogenic switch of cancer-associated fibroblasts via SOCS1/JAK2/STAT3 signaling pathway. *Journal of Experimental & Clinical Cancer Research*.

[28] Yoshida K., Tsuda M., Matsumoto R. (2019). Exosomes containing ErbB2/CRK induce vascular growth in premetastatic niches and promote metastasis of bladder cancer. *Cancer Science*.

[29] Yang H., Zhang H., Ge S. (2018). Exosome-derived miR-130a activates angiogenesis in gastric cancer by targeting C-MYB in vascular endothelial cells. *Molecular Therapy*.

[30] Chen X., Yang F., Zhang T. (2019). MiR-9 promotes tumorigenesis and angiogenesis and is activated by MYC and OCT4 in human glioma. *Journal of experimental & clinical cancer research: CR*.

[31] Wang Z.-F., Liao F., Wu H., Dai J. (2019). Glioma stem cells-derived exosomal miR-26a promotes angiogenesis of microvessel endothelial cells in glioma. *Journal of experimental & clinical cancer research: CR*.

[32] Wortzel I., Dror S., Kenific C. M., Lyden D. (2019). Exosome-mediated metastasis: communication from a distance. *Developmental Cell*.

[33] Aiello N. M., Kang Y. (2019). Context-dependent EMT programs in cancer metastasis. *The Journal of Experimental Medicine*.

[34] Wang L., Yang G., Zhao D. (2020). Correction to: CD103-positive CSC exosome promotes EMT of clear cell renal cell carcinoma: role of remote MiR-19b-3p. *Molecular Cancer*.

[35] Xiao D., Barry S., Kmetz D. (2016). Melanoma cell-derived exosomes promote epithelial-mesenchymal transition in primary melanocytes through paracrine/autocrine signaling in the tumor microenvironment. *Cancer Letters*.

[36] Zhang Q.-Y., Men C.-J., Ding X.-W. (2019). Retracted: Upregulation of microRNA-140-3p inhibits epithelial-mesenchymal transition, invasion, and metastasis of hepatocellular carcinoma through inactivation of the MAPK signaling pathway by targeting GRN. *Journal of Cellular Biochemistry*.

[37] Fu Q., Zhang Q., Lou Y. (2018). Primary tumor-derived exosomes facilitate metastasis by regulating adhesion of circulating tumor cells via SMAD3 in liver cancer. *Oncogene*.

[38] Hashimoto K., Ochi H., Sunamura S. (2018). Cancer-secreted hsa-miR-940 induces an osteoblastic phenotype in the bone metastatic microenvironment via targeting ARHGAP1 and FAM134A. *Proceedings of the National Academy of Sciences of the United States of America*.

[39] Czernek L., Düchler M. (2017). Functions of cancer-derived extracellular vesicles in immunosuppression. *Archivum Immunologiae et Therapiae Experimentalis*.

[40] Wang M., Zhang B. (2021). The immunomodulation potential of exosomes in tumor microenvironment. *Journal of Immunology Research*.

[41] Hayday A. C. (2019). *Γδ* T cell update: adaptate orchestrators of immune surveillance. *Journal of immunology*.

[42] Yin Z., Yu M., Ma T. (2021). Mechanisms underlying low-clinical responses to PD-1/PD-L1 blocking antibodies in immunotherapy of cancer: a key role of exosomal PD-L1. *Journal for immunotherapy of cancer*.

[43] Ye S. B., Li Z. L., Luo D. H. (2014). Tumor-derived exosomes promote tumor progression and T-cell dysfunction through the regulation of enriched exosomal microRNAs in human nasopharyngeal carcinoma. *Oncotarget*.

[44] Chen X., Song E. (2019). Turning foes to friends: targeting cancer-associated fibroblasts. *Nature Reviews Drug Discovery*.

[45] Lei X., Lei Y., Li J. K. (2020). Immune cells within the tumor microenvironment: biological functions and roles in cancer immunotherapy. *Cancer Letters*.

[46] Mao X., Xu J., Wang W. (2021). Crosstalk between cancer-associated fibroblasts and immune cells in the tumor microenvironment: new findings and future perspectives. *Molecular Cancer*.

[47] Szczepanski M. J., Szajnik M., Welsh A., Whiteside T. L., Boyiadzis M. (2011). Blast-derived microvesicles in sera from patients with acute myeloid leukemia suppress natural killer cell function via membrane-associated transforming growth factor- 1. *Haematologica*.

[48] Rong L., Li R., Li S., Luo R. (2016). Immunosuppression of breast cancer cells mediated by transforming growth factor-*β* in exosomes from cancer cells. *Oncology Letters*.

[49] Fabbri M., Paone A., Calore F. (2012). MicroRNAs bind to toll-like receptors to induce prometastatic inflammatory response. *Proceedings of the National Academy of Sciences of the United States of America*.

[50] Ding G., Zhou L., Shen T., Cao L. (2018). IFN-*γ* induces the upregulation of RFXAP via inhibition of miR-212-3p in pancreatic cancer cells: a novel mechanism for IFN-*γ* response. *Oncology Letters*.

[51] Di Xian L. N., Zeng J., Wang L. (2021). LncRNA KCNQ1OT1 secreted by tumor cell-derived exosomes mediates immune escape in colorectal cancer by regulating PD-L1 ubiquitination via MiR-30a-5p/USP22. *Frontiers in cell and developmental biology*.

[52] Chow A., Zhou W., Liu L. (2015). Macrophage immunomodulation by breast cancer-derived exosomes requires Toll-like receptor 2-mediated activation of NF-*κ*B. *Scientific Reports*.

[53] Chen S., Chen X., Qiu J. (2020). Exosomes derived from retinoblastoma cells enhance tumour deterioration by infiltrating the microenvironment. *Oncology Reports*.

[54] Quail D. F., Joyce J. A. (2013). Microenvironmental regulation of tumor progression and metastasis. *Nature Medicine*.

[55] Kaymak I., Williams K. S., Cantor J. R., Jones R. G. (2021). Immunometabolic interplay in the tumor microenvironment. *Cancer Cell*.

[56] Gu J., Qian H., Shen L. (2012). Gastric cancer exosomes trigger differentiation of umbilical cord derived mesenchymal stem cells to carcinoma-associated fibroblasts through TGF-*β*/Smad pathway. *PLoS One*.

[57] Pang W., Su J., Wang Y. (2015). Pancreatic cancer-secreted miR-155 implicates in the conversion from normal fibroblasts to cancer-associated fibroblasts. *Cancer Science*.

[58] Gobbo J., Marcion G., Cordonnier M. (2016). Restoring anticancer immune response by targeting tumor-derived exosomes with a HSP70 peptide aptamer. *Journal of the National Cancer Institute*.

[59] Umezu T., Ohyashiki K., Kuroda M., Ohyashiki J. H. (2013). Leukemia cell to endothelial cell communication via exosomal miRNAs. *Oncogene*.

[60] Zhou W., Fong M. Y., Min Y. (2014). Cancer-secreted miR-105 destroys vascular endothelial barriers to promote metastasis. *Cancer Cell*.

[61] Mashouri L., Yousefi H., Aref A. R., Ahadi A. m., Molaei F., Alahari S. K. (2019). Exosomes: composition, biogenesis, and mechanisms in cancer metastasis and drug resistance. *Molecular Cancer*.

[62] Dong H., Wang W., Chen R. (2018). Exosome-mediated transfer of lncRNA-SNHG14 promotes trastuzumab chemoresistance in breast cancer. *International Journal of Oncology*.

[63] Fornari F., Pollutri D., Patrizi C. (2017). In hepatocellular carcinoma miR-221 modulates sorafenib resistance through inhibition of caspase-3-mediated apoptosis. *Clinical cancer research*.

[64] Wu Q., Yang Z., Nie Y., Shi Y., Fan D. (2014). Multi-drug resistance in cancer chemotherapeutics: mechanisms and lab approaches. *Cancer Letters*.

[65] Lv M.-m., Zhu X.-y., Chen W.-x. (2014). Exosomes mediate drug resistance transfer in MCF-7 breast cancer cells and a probable mechanism is delivery of P-glycoprotein. *Tumour biology: the journal of the International Society for Oncodevelopmental Biology and Medicine*.

[66] Corcoran C., Rani S., O’Brien K. (2012). Docetaxel-resistance in prostate cancer: evaluating associated phenotypic changes and potential for resistance transfer via exosomes. *PLoS One*.

[67] Mostafazadeh N., Samadi N., Kahroba H., Baradaran B., Haiaty S., Nouri M. (2021). Potential roles and prognostic significance of exosomes in cancer drug resistance. *Cell & Bioscience*.

[68] Irie T., Yoshii D., Komohara Y. (2022). IL-34 in hepatoblastoma cells potentially promote tumor progression via autocrine and paracrine mechanisms. *Cancer Medicine*.

[69] Hu Y.-B., Yan C., Mu L. (2019). Exosomal Wnt-induced dedifferentiation of colorectal cancer cells contributes to chemotherapy resistance. *Oncogene*.

[70] Ozawa P. M., Alkhilaiwi F., Cavalli I. J., Malheiros D., de Souza Fonseca Ribeiro E. M., Cavalli L. R. (2018). Extracellular vesicles from triple-negative breast cancer cells promote proliferation and drug resistance in non-tumorigenic breast cells. *Breast Cancer Research and Treatment*.

[71] Levy J. M., Towers C. G., Thorburn A. (2017). Targeting autophagy in cancer. *Nature Reviews. Cancer*.

[72] Huang F., Wang B.-R., Wang Y.-G. (2018). Role of autophagy in tumorigenesis, metastasis, targeted therapy and drug resistance of hepatocellular carcinoma. *World Journal of Gastroenterology*.

[73] Dutta S., Warshall C., Bandyopadhyay C., Dutta D., Chandran B. (2014). Interactions between exosomes from breast cancer cells and primary mammary epithelial cells leads to generation of reactive oxygen species which induce DNA damage response, stabilization of p53 and autophagy in epithelial cells. *PLoS One*.

[74] Hamurcu Z., Delibaşı N., Geçene S. (2018). Targeting LC3 and Beclin-1 autophagy genes suppresses proliferation, survival, migration and invasion by inhibition of Cyclin-D1 and uPAR/Integrin *β*1/Src signaling in triple negative breast cancer cells. *Journal of Cancer Research and Clinical Oncology*.

[75] Mele L., del Vecchio V., Liccardo D. (2020). The role of autophagy in resistance to targeted therapies. *Cancer Treatment Reviews*.

[76] Panigrahi G. K., Deep G. (2017). Exosomes-based biomarker discovery for diagnosis and prognosis of prostate cancer. *Frontiers in bioscience (Landmark edition)*.

[77] Kok V. C., Cheng-Chia Y. (2020). Cancer-derived exosomes: their role in cancer biology and biomarker development. *International Journal of Nanomedicine*.

[78] Kumar D., Gupta D., Shankar S., Srivastava R. K. (2015). Biomolecular characterization of exosomes released from cancer stem cells: possible implications for biomarker and treatment of cancer. *Oncotarget*.

[79] Sohn W., Kim J., Kang S. H. (2015). Serum exosomal microRNAs as novel biomarkers for hepatocellular carcinoma. *Experimental & molecular medicine*.

[80] Qu Z., Wu J., Wu J. (2017). Exosomal miR-665 as a novel minimally invasive biomarker for hepatocellular carcinoma diagnosis and prognosis. *Oncotarget*.

[81] Ge Y., Mu W., Ba Q. (2020). Hepatocellular carcinoma-derived exosomes in organotropic metastasis, recurrence and early diagnosis application. *Cancer Letters*.

[82] Cui S., Cheng Z., Qin W., Jiang L. (2018). Exosomes as a liquid biopsy for lung cancer. *Lung cancer*.

[83] Silva J., Garcia V., Zaballos A. (2011). Vesicle-related microRNAs in plasma of nonsmall cell lung cancer patients and correlation with survival. *The European Respiratory Journal*.

[84] Madhavan B., Yue S., Galli U. (2015). Combined evaluation of a panel of protein and miRNA serum-exosome biomarkers for pancreatic cancer diagnosis increases sensitivity and specificity. *International Journal of Cancer*.

[85] Liu H., Qiao S., Fan X., Gu Y., Zhang Y., Huang S. (2021). Role of exosomes in pancreatic cancer (review). *Oncology Letters*.

[86] Wang J., Wang X., Song Y. (2019). Circulating noncoding RNAs have a promising future acting as novel biomarkers for colorectal cancer. *Disease Markers*.

[87] Filella X., Foj L. (2016). Prostate cancer detection and prognosis: from prostate specific antigen (PSA) to exosomal biomarkers. *International Journal of Molecular Sciences*.

[88] Lakshmi S., Hughes T. A., Priya S. (2020). Exosomes and exosomal RNAs in breast cancer: a status update. *European journal of cancer (Oxford, England: 1990)*.

[89] Jia Y., Chen Y., Wang Q. (2017). Exosome: emerging biomarker in breast cancer. *Oncotarget*.

[90] Li M., Zhou Y., Xia T. (2018). Circulating microRNAs from the miR-106a-363 cluster on chromosome X as novel diagnostic biomarkers for breast cancer. *Breast Cancer Research and Treatment*.

[91] Joyce D. P., Kerin M. J., Dwyer R. M. (2016). Exosome-encapsulated microRNAs as circulating biomarkers for breast cancer. *International Journal of Cancer*.

[92] Revenfeld A. L., Bæk R., Nielsen M. H., Stensballe A., Varming K., Jørgensen M. (2014). Diagnostic and prognostic potential of extracellular vesicles in peripheral blood. *Clinical Therapeutics*.

[93] Resnick K. E., Alder H., Hagan J. P., Richardson D. L., Croce C. M., Cohn D. E. (2009). The detection of differentially expressed microRNAs from the serum of ovarian cancer patients using a novel real-time PCR platform. *Gynecologic Oncology*.

[94] Mengual L., Lozano J. J., Ingelmo-Torres M., Gazquez C., Ribal M. J., Alcaraz A. (2013). Using microRNA profiling in urine samples to develop a non-invasive test for bladder cancer. *International Journal of Cancer*.

[95] Barreiro K., Huber T. B., Holthofer H. (2020). Isolating urinary extracellular vesicles as biomarkers for diabetic disease. *Methods in molecular biology*.

[96] Husna A. A., Rahman M. M., Lai Y.-C. (2021). Identification of melanoma-specific exosomal miRNAs as the potential biomarker for canine oral melanoma. *Pigment Cell & Melanoma Research*.

[97] Vaidya M., Bacchus M., Sugaya K. (2018). Differential sequences of exosomal NANOG DNA as a potential diagnostic cancer marker. *PLoS One*.

[98] Lan B., Zeng S., Grützmann R., Pilarsky C. (2019). The role of exosomes in pancreatic cancer. *International Journal of Molecular Sciences*.

[99] Xiao Y., Zhong J., Zhong B. (2020). Exosomes as potential sources of biomarkers in colorectal cancer. *Cancer Letters*.

[100] Lorenc T., Klimczyk K., Michalczewska I., Słomka M., Kubiak-Tomaszewska G., Olejarz W. (2020). Exosomes in prostate cancer diagnosis, prognosis and therapy. *International Journal of Molecular Sciences*.

[101] Shi F., Wu H., Qu K. (2018). Identification of serum proteins AHSG, FGA and APOA-I as diagnostic biomarkers for gastric cancer. *Clinical Proteomics*.

[102] Liang B., Peng P., Chen S. (2013). Characterization and proteomic analysis of ovarian cancer-derived exosomes. *Journal of Proteomics*.

[103] Wang Y.-T., Shi T., Srivastava S., Kagan J., Liu T., Rodland K. D. (2020). Proteomic analysis of exosomes for discovery of protein biomarkers for prostate and bladder cancer. *Cancers*.

[104] Sun W., Luo J.-d., Jiang H., Duan D. D. (2018). Tumor exosomes: a double-edged sword in cancer therapy. *Acta Pharmacologica Sinica*.

[105] Dawood S., Austin L., Cristofanilli M. (2014). Cancer stem cells: implications for cancer therapy. *Oncology*.

[106] Zhang Z., Li X., Sun W. (2017). Loss of exosomal miR-320a from cancer-associated fibroblasts contributes to HCC proliferation and metastasis. *Cancer Letters*.

[107] Ristorcelli E., Beraud E., Verrando P. (2008). Human tumor nanoparticles induce apoptosis of pancreatic cancer cells. *FASEB journal*.

[108] Zheng R., du M., Wang X. (2018). Exosome–transmitted long non-coding RNA PTENP1 suppresses bladder cancer progression. *Molecular Cancer*.

[109] Xu G., Zhang B., Ye J. (2019). Exosomal miRNA-139 in cancer-associated fibroblasts inhibits gastric cancer progression by repressing MMP11 expression. *International Journal of Biological Sciences*.

[110] Lu J., Liu Q.-H., Wang F. (2018). Exosomal miR-9 inhibits angiogenesis by targeting MDK and regulating PDK/AKT pathway in nasopharyngeal carcinoma. *Journal of experimental & clinical cancer research: CR*.

[111] Lee Y. T., Tan Y. J., Oon C. E. (2018). Molecular targeted therapy: treating cancer with specificity. *European Journal of Pharmacology*.

[112] Kijanka M., Dorresteijn B., Oliveira S., van Bergen en Henegouwen P. M. P. (2015). Nanobody-Based Cancer Therapy of Solid Tumors. *Nanomedicine (London, England)*.

[113] Batrakova E. V., Kim M. S. (2015). Using exosomes, naturally-equipped nanocarriers, for drug delivery. *Journal of Controlled Release: Official Journal of the Controlled Release Society*.

[114] Harrell C. R., Jovicic N., Djonov V., Arsenijevic N., Volarevic V. (2019). Mesenchymal stem cell-derived exosomes and other extracellular vesicles as new remedies in the therapy of inflammatory diseases. *Cell*.

[115] Barile L., Vassalli G. (2017). Exosomes: therapy delivery tools and biomarkers of diseases. *Pharmacology & Therapeutics*.

[116] Ha D., Yang N., Nadithe V. (2016). Exosomes as therapeutic drug carriers and delivery vehicles across biological membranes: current perspectives and future challenges. *Acta pharmaceutica Sinica. B*.

[117] Haney M. J., Klyachko N. L., Zhao Y. (2015). Exosomes as drug delivery vehicles for Parkinson's disease therapy. *Journal of Controlled Release: Official Journal of the Controlled Release Society*.

[118] Pan S., Zhang Y., Huang M. (2021). Urinary exosomes-based engineered nanovectors for homologously targeted chemo- chemodynamic prostate cancer therapy via abrogating EGFR/AKT/NF-kB/IkB signaling. *Biomaterials*.

[119] Qi H., Liu C., Long L. (2016). Blood exosomes endowed with magnetic and targeting properties for cancer therapy. *ACS Nano*.

[120] Lamichhane T. N., Jeyaram A., Patel D. B. (2016). Oncogene knockdown via active loading of small RNAs into extracellular vesicles by sonication. *Cellular and Molecular Bioengineering*.

[121] Yong T., Zhang X., Bie N. (2019). Tumor exosome-based nanoparticles are efficient drug carriers for chemotherapy. *Nature Communications*.

[122] Qiao L., Hu S., Huang K. (2020). Tumor cell-derived exosomes home to their cells of origin and can be used as Trojan horses to deliver cancer drugs. *Theranostics*.

